# Public transport: lessons learned by the sector through the COVID-19 pandemic

**DOI:** 10.1186/s12889-023-16062-3

**Published:** 2023-10-02

**Authors:** Nicola Gartland, Anna Coleman, Bernadine Farrell, David Fishwick, Sheena Johnson, Martie van Tongeren

**Affiliations:** 1 Centre for Occupational and Environmental Health, School of Health Sciences, University of Manchester, Manchester, UK; 2 Manchester Academic Health Science Centre, Manchester, UK; 3 The Thomas Ashton Institute for risk and regulatory research, University of Manchester, Manchester, UK; 4 Centre for Workplace Health, Health and Safety Executive Science and Research Centre, Harpur Hill, Buxton, UK; 5 Alliance Manchester Business School, University of Manchester, Manchester, UK

**Keywords:** COVID-19, Coronavirus, Control measures, Public transport, Transmission, Longitudinal, Reflection

## Abstract

**Background:**

The COVID-19 pandemic had a significant impact on the operations and functionality of the public transport sector in the UK. This paper reflects on the experience of this sector through the pandemic period, and considers recommendations for any future mitigations required for either new COVID-19 waves or a different public health emergency.

**Methods:**

Semi-structured interviews were carried out with public transport experts, organisational leaders, workers and passengers in two phases: Phase 1 from January to May 2021, and Phase 2 from December 2021 to February 2022. Interviews were analysed thematically.

**Results:**

Using the ‘What? So What? Now What?’ reflective model, ideas are drawn out to describe (a) what changes occurred, (b) what effects these changes had on service provision as well as perceptions of risk and mitigation and (c) what lessons have been learned and how these findings can feed into pandemic preparedness for the future. Respondent reflections focussed on the importance of communication, leadership, and maintaining compliance.

**Conclusions:**

The wealth of experience gained through the COVID-19 pandemic in the public transport sector is extremely valuable. Through reflection on this experience, specific recommendations are made relating to these factors, covering: maintaining links across industry, access to information and data, understanding of mitigation effectiveness, improving messaging, challenges of behavioural mitigations, and clear lines of accountability. The recommendations made on the basis of this reflective process will help to improve public health strategy within the public transport sector.

## Introduction

Public transport has been a high profile sector since the start of the COVID-19 pandemic in March 2020. It was identified as a potential high risk for COVID-19 transmission for both workers and passengers [1, 2], but was also an essential service that was supported financially by the UK government in order to maintain provision even with very low passenger numbers. Before the start of the first national lockdown on March 26^th^ 2020, the UK Prime Minister said “now is the time for everyone to stop non-essential contact and to stop all unnecessary travel” [3]. Therefore, considerations around travel have been at the forefront of the pandemic response from the beginning.

**Fig. 1:**
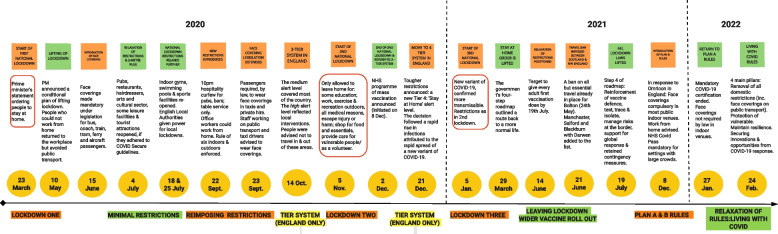
Timeline of pandemic, relevant to public transport sector. Sources: www.gov.uk (accessed 17 March 2023); https://www.instituteforgovernment.org.uk/charts/uk-government-coronavirus-lockdowns (accessed 17 March 2023)

COVID-19 regulations were developed quickly by the UK government, and implemented across the public transport sector. The UK government published general transport and travel guidance in April 2020 [4], and this was followed by more specific guidance for transport operators in May 2020 [5]. Since first publication, this guidance has been updated over 30 times. Transport guidance was issued separately for England, Northern Ireland, Scotland and Wales. Guidance for passengers was also published initially in May 2020, and has been updated over 45 times since first publication [6]. Guidance for taxis and private hire vehicles (PHVs) was published later in November 2020 [7]. Figure 1 shows key dates for the public transport sector during the pandemic, in the context of other significant COVID-19 milestones.

The transport sector worked hard through the pandemic to understand the risks of SARS-CoV-2 (the virus that causes COVID-19) and implement ever-changing guidance to mitigate transmission of the virus [8, 9]. Quantifying and mitigating these risks has been a challenge, and some guidance and mitigations have had unintended consequences; for example, reducing employee contact at work was reported to have resulted in increased staff isolation and reduced ridership impacted on job satisfaction for some workers [10]. It is appropriate at this point to reflect on the process the sector has been through, and collate the lessons learned through this experience.

Reflective practice, particularly for crisis management within organisations, is a valuable tool which can lead to effective learning and increased resilience in the future [11, 12]. The importance of reflective learning has been highlighted recently in a report from the House of Commons Committee of Public Accounts, which stated “the government will need to learn lessons from its preparations for and handling of these risks to improve the identification, assessment and response to future risks that affect the whole system”(pg. 4; [13]). The planned UK COVID-19 Inquiry will also review the preparations and response to COVID-19 in terms of travel, borders and interventions for social distancing [14].

Reports such as the ‘Coronavirus: Lessons learned to date’ report from the House of Commons Health and Social Care and Science and Technology Committees have drawn out some of the wider lessons learned through the pandemic [15]. This reported a large number of recommendations, including the need for established protocols for sharing data between public bodies, greater operational competence in the establishment of test and trace programmes, and better accessibility to health advice and outreach programmes tailored to cultural contexts in different communities.

However, reports have not focussed on the lessons learned specifically related to public transport yet. Transport Focus (an independent Watchdog for transport users) concentrated on the passenger perspective and published key lessons learned in January 2021 [16]. This included important reflections regarding the continued use of mitigations to give passengers confidence to make journeys, and the need for active promotion of the sector in order to attract passengers back when appropriate.

Further lessons can now be drawn from this sector. Taking a longitudinal perspective, data was collected in two phases to capture the many changes in policy and mitigation measures, as well as changing public perceptions within the fast moving nature of the pandemic. This longitudinal approach is valuable for facilitating critical appraisal of the decisions made through the course of the pandemic, which is necessary to inform future responses to COVID-19 or other public health emergencies. In parallel, important work has been published considering future public transport policy and balancing the needs of the sector with changes in mobility that have occurred as a result of the pandemic [17, 18].

Reflections have been framed using the simple ‘What?, So What?, Now What?’ model of reflection which was developed by Rolfe et al. and Driscoll [19, 20]. This model has been extensively used to promote professional learning and development by getting the most out of experiential learning, particularly in the areas of healthcare and nursing [21]. However, the use of reflection at the organisational level has also been argued to foster organisational learning [22–25]. This reflective model has the benefit of being adaptable to different situations, and thus it has been applied here to reflect on the management of COVID-19 within the public transport sector through the pandemic.

### Aim

This research aimed to provide a longitudinal perspective on the development of understanding of transmission risks and mitigation as well as the introduction and removal of COVID-19 regulations within the sector. Data from two phases of data collection was used to address what went well, what issues arose, and what could be improved upon. Drawn from this qualitative analysis, recommendations are presented to inform future waves of COVID-19, future pandemics and other public health emergencies.

The aim of this study was to answer the following questions using data collected across two phases of the research:

1. What… is the context of the situation, and what was done to help resolve the situation?
2. So what… were the implications of the changes made, and what does the experience tell us about the response to the situation? What is our new understanding of the situation?
3. Now what… should be done in the future to address similar future risks?

## Method

A qualitative ‘deep dive’ approach was employed to explore the perceptions of SARS-CoV-2 transmission risk and the effectiveness of risk mitigation measures to control transmission within the public transport sector, from the perspective of a range of public transport stakeholders. Major knowledge gaps were also explored. A partnership with a wide group of stakeholders was established at the outset of this work (involving experts, organisational leaders and regulators), and a significant informal engagement exercise was carried out to inform the design of this project. To make the research feasible and due to their common usage [26], bus and rail transport in the UK was the initial focus; although stakeholders from the taxi / tram sectors were recruited in later stages of data collection.

Semi-structured interviews were carried out in two phases: Phase 1 from January to May 2021, and Phase 2 from December 2021 to February 2022. In both phases, the views of a range of stakeholders were sought, including: experts, organisational leaders, workers and passengers. The recruitment strategy was purposive; connections made at the initial engagement/information gathering stage (October – December 2020) were used to identify appropriate participants for Phase 1 data collection, and a snowballing method was also utilised to recruit more widely through this network. Ethics approval was granted from the University of Manchester Proportionate Review Committee (Ref: 2021–10,535-17,496). Informed consent was obtained from all participants.

### Phase 1

Recruitment to Phase 1 was staged, with interviews initially held with experts and organisational leaders who then provided a route into passenger and employee groups [8]. Forty-seven interviews were carried out between late January and May 2021, with experts (research, policy, industry, *N* = 17) organisational representatives (including unions, *N* = 13), workers (*N* = 5) and passengers (*N* = 12). See Table 1. For further details, see Coleman et al. [8].

**Table 1:** Summary of recruitment for Phase 1 and 2

Interviewee type	Phase 1 (Jan-May 2021)	Phase 2 (Dec 2021-February 2022)
Expert	17	5
Organisational Leader	13	5
Worker	5	2
Passenger	12	5
**Total**	**47**	**17**

Quotes are used to illustrate findings, where appropriate. Respondent and organisational identity are kept confidential by use of identification codes, prefixed: *EX* experts, *OL* organisational leaders, *W* workers, or *P* passengers.

The qualitative interviews were carried out by two skilled researchers using videoconferencing platforms (Zoom / TEAMS) lasting an average of 60 min. Interviews were recorded with permission of the respondents, were professionally transcribed, and analysed thematically. Global, organising, and basic themes were identified, to describe the perceptions of risk and risk mitigation effectiveness, experience of changes introduced to the public transport sector to reduce transmission of SARS-CoV-2, and considerations for how the sector will move forward in the future [8].

### Phase 2

Phase 2 was designed to provide updated data on the views recorded in Phase 1, to deepen the understanding of how the perceptions of the risk of SARS-CoV-2 transmission and of the effectiveness of mitigation measures introduced in the public transport sector in the UK had developed through the pandemic in the context of changing circumstances/regulations [9]. Participants were asked about:

- general thoughts on the current situation (winter 2021–22) including risk perceptions;
- changes in risk perception and mitigation since the prior interview (between 7–12 months) focussing primarily on July 2021 onwards (when most mitigations were reduced in England);
- changes in risk perception and mitigation since rules changed again in December 2021 (Omicron variant);
- the evolution of strategies undertaken to reduce the spread of COVID-19.

Interviews were carried out, with a subsample of the original 47 participants, between mid-December 2021 and early February 2022 (see Table 1). The aim was to speak to 20 of the original sample (5 from each respondent category), to include a broad range of respondents (jobs, roles, transport usage), modes of travel, and geographical location. For further details, see Coleman et al. [9].

Phase 2 interviews were carried out by the same two qualitative researchers using videoconferencing platforms (Zoom / Teams), were transcribed verbatim and analysed thematically.

The results have been structured using the ‘What?, So What?, Now What?’ reflective model [19, 20]. Using this model, this study draws on stakeholder reflections on the management of risk within the sector over the course of the pandemic, and identify valuable lessons learned specific public transport through the process.^1^

## Results

A full account of Phase 1 and Phase 2 findings can be found in Coleman et al. [8, 9]. The findings from these reports have been summarised and reflected upon here to identify the lessons learned through the COVID-19 pandemic.

### WHAT has happened within the public transport sector over the course of the pandemic?

#### Context and information sources

In Phase 1, all experts and organisational leaders described significant changes to their work in the previous 12 months, including shifting the focus of work to address COVID-19 directly, or working to develop or implement new policies or guidance within the many diverse public transport spaces. All described challenges arising from the rapidly changing environment and the need to gain the right knowledge to inform decision making in an accurate and timely way.

Workers also discussed significant changes to their roles and responsibilities at Phase 1. Frontline staff experienced some periods at home (either working on call, or stood down) and periods of furlough. Staff with medical conditions that might have put them at additional risk were particularly protected from frontline work. These changes were generally reported to be acceptable and necessary; however, there was an associated impact on morale. For further details, please see Gartland et al. [10].

*“I’ll just be in First Class by myself, the other person will be up in Standard by themselves. And that has been a big change [ … ] and not really in a good way either because, you’re just alone all day and you don’t have that camaraderie [ … ] and the support from your colleagues, you don’t see them.” (W4, Phase 1)*

Organisational leaders and experts (industry) frequently discussed the benefits of joint working across the sector in order to facilitate information and knowledge sharing; this was achieved through both formal and informal forums. Joint working was reported to help develop consistent and clear messaging across companies. It was most common for companies to group together by mode of transport. For example, the Confederation for Passenger Transport (CPT) facilitated this for the bus sector, while a new Rail industry COVID-19 forum was established via the Rail Delivery Group (RDG).

All passengers interviewed reported a significant change to travel patterns, including reducing use of public transport, changes to mode of travel, and using public transport for essential travel only. Workers and organisational leaders also discussed low passenger numbers through the pandemic and while ridership increased between Phase 1 and Phase 2, usage was still lower than pre-pandemic levels.

By Phase 2, those working in the public transport sector suggested that less of their time was consumed with responding directly to the pandemic. This was due to a combination of being better able to respond quickly to change having gained knowledge and understanding of responses required (‘toolkit’); systems, partnerships and fora being operational to aid a sector response; and a perceived lower risk of disease transmission.

Virus variants also changed through the data collection periods; during Phase 1, the Alpha variant was firstly most prevalent, but concerns then grew about the Delta variant. By Phase 2, the Omicron variant had become the dominant variant in the UK. An additional contextual factor was the changing nature and extent of the vaccination programme. During Phase 1, the initial vaccine programme was being rolled out. By Phase 2, reliance on vaccination as a mitigation measure was increasing, and many individuals had received three doses.

*“The new variant, it’s another new variant, we’ve just dealt with it like we have every other one, to be honest. I’ve tried to redirect the forces of what we do away from just being COVID, which is what it was at one point, to going back to regulating the health and safety operation of the railway.” (EX18, Phase 2)*

##### Sources of information

In both phases, experts and organisational leaders were asked about sources of information that were available and accessible to help with their decision making in relation to COVID-19. In both phases of the research the organisational leaders stated that they went to sources they implicitly trusted and were generally backed by public health professionals and scientists. See Table 2 for commonly mentioned sources.

**Table 2:** Details of sources mentioned by respondents

Category	Sources	Details
Government departments	Department for Transport (DfT)	Official safety guidance, advice, information
	Devolved Governments	
	Health and Safety Executive (HSE)	
Government Public Health	Health Security Agency (UKHSA; formerly Public Health England, PHE)	Data on infection rates, different virus variants
	Devolved Public Health organisations	
	Local Authorities (LAs)	
Ongoing research	Real-time Assessment of Community Transmission (REACT)	Data on infection and transmission rates, effectiveness of face coverings, cleaning, ventilation, role of vaccines, transmissibility of new variants
	TRACK / VIRAL studies	
	Office for National Statistics (ONS)	
	ZOE app	
	Rail Safety and Standards board (RSSB)	
	Academic papers on mitigation effectiveness	
Public Transport Sector	Fora (formal and informal)	Sector experience and knowledge sharing
	Regulators/ industry bodies,	
	Unions	
Passenger and Worker Feedback	Organisational/regional or national scale, discussions, surveys, social media	Acceptability of mitigations, perceptions of safety, and issues
	Other sources (e.g. Transport Focus surveys)	

Source: Coleman et al. [8]

Experts and organisational leaders also reported formal links with regulatory and oversight bodies such as DfT, UKHSA (formerly PHE) and LAs (in relation to public health), as well as the HSE, mode specific regulators (e.g. Office of Rail and Road (ORR)) and transport representative bodies (e.g. CPT).

Passengers who were using transport during the pandemic (referred to here as ‘current passengers’) suggested they received enough information about travel rules from general and company-specific communications, and generally knew where to find information. However, they also said that sometimes it was confusing, especially when guidance from different sources appeared to be contradictory. Lapsed passengers (those who had used public transport before the pandemic, but stopped using any during the pandemic) tended to report the need to avoid public transport or only use it if there are no other options:

*“I guess from a public transport perspective, the message that I’ve picked up, and it may be the wrong message but it’s just the one about, you know, don’t use it unless you really have to or try to avoid it, you know, that message hasn’t really changed in my mind since.” (P12, lapsed passenger, Phase 1)*

#### Risk mitigations

Table 3 shows mitigation measures implemented in the public transport sector to mitigate viral transmission, as raised by the organisational leaders, Union representatives and experts during Phase 1 (see Table 3). While no *new* mitigations had been implemented by Phase 2, respondents discussed having a better understanding of transmission routes and mitigations and tackling airborne transmission became more important (e.g. ventilation).

**Table 3:** Phase 1 Mitigation measures (Experts and organisational leaders)

Mitigation (Listed alphabetically)	Detail
***For passengers / workers***	
Capacity on transport	Numbers of services, numbers of seats accessible etc. to avoid overcrowding, reservation only services
Enhanced cleaning regimes	More regular, focus on touch points, different cleaners, antiviral surface treatments, fogging machines
Face coverings (often referred to by respondents as masks)	Face coverings were mandatory^a^ on public transport for passengers and workers in specified contexts
Hand sanitiser provision	Provision by organisations for workers / passengers
Social distancing	1 m + and 2 m, seats closed off, in places where staff gather, limiting worker and passenger interaction, signage, one way systems
Stations/bus stops adaptations	Social distanced queues, one way systems, new signage / posters
Technology	Contactless tickets/payments, apps, virtual training
Temperature screening	Passengers/workers
Ventilation	Increased air circulation, keeping windows open (signage)
***Additional for passengers***	
No eating/drinking/free papers on public transport	Less touch points / litter
***Additional for workers***	
Adaptions in vehicle	Screens, money shoots, one way systems
Campaigning for early vaccinations	Campaigning as key workers (by unions and employers)
Clinically vulnerable staff—shielding	Shielding if worker or family needs, pregnant workers
PPE/gloves	Visors, masks (grade higher than face covering) and gloves
Staff testing and isolation	Lateral flow/PCR^b^, private testing
Staff working from home/furlough	Taking staff away from risk
Training (staff)	Keeping safe, keeping passengers safe, challenging behaviour
Work bubbles	Close proximity, shift pattern staggering, in-person training

^a^Except for those ‘exempt’ (or claiming to be exempt), which is a point of difficulty for implementation

^b^PCR polymerase chain reaction

Source: Coleman et al. [8]

In Phase 2, mitigations had become less consistent across the sector and geographically (across devolved countries) due to the removal of many government regulations. However, many of the public transport companies had chosen to keep some mitigations in place, despite the relaxing of rules as they believed they had helped to keep both workers and passengers safer.

From the interviews conducted in Phase 2, many companies were still using enhanced cleaning regimes and encouraged face covering usage (workers and passengers), and predicted these being in place for the longer term. Social distancing was seen as unworkable, for most companies and modes of transport (although easier where seats were bookable like long distance rail or coaches), especially when footfall is so important to revenue. One-way systems were also removed by most, and were only kept where this improved flow more generally. Many stressed that ventilation was important, with some suggesting the vehicles had very good turnover of airflow (trams) and others saying that keeping windows blocked slightly open (buses) even in winter was essential. There was also discussion about long-term moves to improve ventilation, for example through the introduction of new filters on trains, but this was seen as something that would gradually be introduced.

*“So we retained those measures in place and we felt they were sensible in order to minimise workplace transmission. We didn’t go the whole hog in terms of removing restrictions. When Omicron came along at the end of last year the only additional thing that we had to do was reintroduce one-way systems and re-instigate working from home for certain individuals.” (OL12, Phase 2)*

### SO WHAT have been the implications of the implementation of mitigation measures?

#### Perceptions of risk

Broadly, in both phases of the research, the perception of risk on public transport by all stakeholders was deemed an acceptable and small risk. However, some recognised that the level of risk could vary with factors including time of day, location of travel, duration of travel, rural vs urban settings, type of vehicle, and other passengers. It was also pointed out that public transport is used to visit a range of destinations, and that the risk of transmission in those destinations may be equal to or greater than the risk on public transport itself.

#### Effectiveness of mitigations

Respondents provided valuable reflections on effectiveness of mitigations during Phase 1, when most mitigations were in place. At that time, most suggested it was difficult to tell which mitigation strategies were effective as the relative risk of the three transmission routes was unknown, and also because mitigations were introduced concurrently. Also, importantly, it was suggested that face coverings provided effective mitigation because they help to reduce transmission through all transmission routes and were argued to be a sensible public health mitigation wider than COVID-19.

*“The issue we keep hitting in the rail industry, is we don’t know how good any mitigation is. [ … ] we don’t know how many transmissions have happened on rail. [ … ] And then we don’t know what kind of proportion of infections are caused by the aerosol route, the large droplet route, or the surface contact, people touching infected surfaces. So, we don’t know how that splits down, and then that impacts all the different mitigations.” (EX6, Phase 1)*

The rapid introduction of multiple mitigations simultaneously also made it difficult to assess effectiveness, as it could not be determined if reductions in transmission were associated with particular mitigations or the particular *combination* of mitigations. Furthermore, the overlaying of mitigations without sufficient evaluation was also proposed to potentially impact their effectiveness, where one mitigation might actually inhibit the effectiveness of another.

*“We've done a massive amount on cleaning and hygiene [ … ] What we tended to do is we've overlaid a lot of cleaning on our normal cleaning regime, and we’re actually now get to the point of thinking, actually we've layered stuff on top and it's not always necessarily the most effective way of doing it.” (OL4, Phase 1)*

Some experts reported that effectiveness of mitigations was related to acceptability and behavioural compliance. For example, behavioural mitigations could be challenging to enact if they caused discomfort, such as keeping vehicle windows open in the winter or developing skin irritation from hand sanitiser.

*“There’s a difference between what would be most effective and what would be most tolerated by people. So, you could put in very effective measures, but people would, maybe, stop using the bus or stop abiding by them. So, effectiveness equals, the measure of compliance, if you like.” (EX16, Phase 1)*

Workers and leaders spoke about some of the challenges of applying the new rules, particularly in confined spaces and with long-standing colleagues. This was partially attributed to trust between colleagues, which was difficult to reconcile with some mitigations.

*“So in terms of on train crew, because they're in a smaller environment, they sometimes forget that social distancing still needs to be adhered to. And I think because they are really good friends and it's like sometimes going to work is like a day out for them really.” (W1, Phase 1)*

In the case of increased cleaning regimes, at Phase 1 these were perceived to be effective for mitigating fomite transmission; some companies tested surfaces with swabs to sample for viral material and when tests were negative this was assumed to reflect effective cleaning practices. Enhanced cleaning and hygiene was also an important visual reassurance that gave passengers increased confidence in their safety on public transport. By Phase 2, airborne transmission was generally considered the primary route of transmission; however, cleaning regimes were largely maintained because of the positive impact on passenger confidence.

*“The hygiene theatre makes them feel happy even though it doesn’t probably do very much.” (EX8, Phase 1)*

Many of our respondents (especially organisational leaders) appeared to assume that as there had been relatively few observed outbreaks within their workforce, the mitigations in place were effective and public transport companies had done everything they could to keep workers and passengers safe. The term ‘COVID-safe’ was used by some respondents, but others suggested it was a misleading label because of the difficulties of removing all risk of transmission.

*“Yeah, so I think since June or July [2020] when we brought the safe systems of work in, generally the indications are that they’ve been working very satisfactorily. [ … ] I am satisfied that our safe systems of work are really doing their job in terms of limiting transmission, both customers and employees.” (OL5, Phase 1)*

#### Barriers and facilitators for the implementation of mitigations

In addition to reflections on the effectiveness of mitigations introduced, respondents also provided reflections on the barriers and facilitators which influenced the introduction of mitigations in the sector. A full list of facilitators and barriers is available in the report published by Coleman et al. [8].

##### Barriers

The barriers identified were multifaceted, and ranged from the communication of information regarding advised mitigations, individual compliance with behavioural mitigations, issues with capacity on vehicles and balancing running costs, pressures on the workforce due to COVID-related absences, and a lack of transmission data within the sector. These barriers are explored in detail in the sections below.

*Interpreting guidance*

For transport providers, interpreting messages from Government and advisory bodies was not always straightforward. The messages were subject to quick change and sometimes were not easily practicable or detailed enough, and often arrived with very short notice. Particular issues discussed by respondents included: leaving guidance open to interpretation, messages could induce fear in employees and customers, inconsistencies between interpretations of guidelines between different government departments leading to requests to change risk assessments, lack of clarity on details (e.g. move from 2 m social distancing to 1 m + mitigation, implications of more transmissible variants), arrangements for self-isolation payments which could promote presenteeism (workers), and differences in advice issued by devolved Governments (particularly for companies that provided services that crossed country boundaries). The need for clarification from Government was discussed, especially at the outset or when circumstances changed. Respondents explained that moving from published guidance to operational practice could be complex and time consuming.

*“This is about the physical partition, and they’ve worded it in such a way as it gives them carte blanche to interpret things differently. And we have had this out with them, and they’re devils for doing this, to make sure that they don’t leave themselves in a difficult position. And you could interpret that on school vehicles each seat has to be encased in Perspex floor to ceiling.” (OL14, Phase 1)*

However, workers generally reported their satisfaction with communications from employers (once digested and re-communicated by companies) about the pandemic and the implementation of mitigation strategies.

*“ … they send regular updates via email, so any changes to any restrictions, any policies, and any ways of working they are clearly detailed in an email to everyone across the business. We have an intranet site, which one of our pages is dedicated to Coronavirus. [ … ] But the company are really, really good, they are very detailed in any changes that do happen.” (W1, Phase 1)*

The majority of workers and passengers (both current and lapsed passengers) believed travel guidance during the pandemic was easy to understand. However, complications arose when transport guidance and rules differed to the general pandemic guidance (e.g. requirement for face coverings on public transport vs no similar requirement in shops). Among passengers who had continued to use public transport during the pandemic, there was some concern about the practicality of some guidance. For example, instructions to use quieter stations or to wait for the next service if the current one was busy. If journey time mattered, this was perceived as unhelpful (e.g. work commute / medical appointments).

*“You can’t [wait for the next service], ‘cause that’s in conflict with some of the major train companies, where you have to have booked a seat. And it’s impractical if you live in an area where there’s only an hourly service. [ … ] And I think to say don’t eat or drink on a journey that might last for three hours, is a bit unrealistic frankly, I mean most people want at least a drink of water in that time. [ … ] And also, not touching a surface, I mean, it’s unrealistic, you can’t walk around with your hands in the air on a train, or getting on a bus, because you’d fall over.” (P8, current passenger, Phase 1)*

*Compliance with behavioural mitigations*

Across both Phases, the primary barriers discussed were at least in some way related to behaviour (e.g. wearing a face covering, social distancing, sanitising, opening windows, etc.), and continued behavioural adherence at an individual level was essential.

Broadly, compliance was seen as high across the different behaviours, especially at the start of the pandemic. However, it was noted that even small numbers of observed incidents would be salient and thus could have a large impact on perceptions of risk. Over time, and with the reintroduction of the face covering mandate in December 2021, adherence was harder to maintain with certain populations; some workers and passengers were resistant to the idea of wearing face coverings again. This was attributed by some to the increased levels of vaccination and virus exposure in the population.

*“It doesn’t take a lot and we’ve seen stubborn significant minorities on some of those measures, like the other passenger behaviour, coming through from some of the satisfaction scores. It’s like... that doesn’t take a lot of that to, sort of, percolate through and create a situation where people don’t think it’s a safe environment.” (EX9, Phase 1)*

In order to facilitate social distancing, organisational leaders described companies monitoring and calculating expected capacity and demand, or alternatively using proxy measures (e.g. vehicle loading, seat reservations). For workers in the sector, regular observational checks were made, but employees were generally trusted to comply.

Complex cleaning regimes were implemented, at time and financial cost. Some challenges were identified where employees were expected to carry out cleaning between shifts. Within many organisations, management entrusted employees with cleaning mitigations in their own work spaces, but one organisational leader noted that this had led to low compliance with the advised cleaning protocol between shifts. This highlights the difficulties of implementing new behaviours in established organisational structures, where interpersonal dynamics between colleagues may play a role in compliance with the new behaviours.

*“So, I think they felt they were insulting the person they were taking over from.” (OL15, Phase 1)*

The attribution of responsibility for policing and challenging of passengers who were not compliant with behavioural mitigations proved challenging. Divergent views were held by those working in the sector compared to passengers. From an organisational leader perspective, public transport companies were only able to ‘advise and encourage’. Enforcement was reported to be the responsibility of transport police (trains) or the police. Workers found this difficult; they spoke about needing to challenge passengers but having no way of enforcing the rules, and policing colleagues was described as especially difficult. There was an interesting contrast in perspectives here with passengers, as they often reported expecting the rules to be enforced by workers. This was an emotive topic as both passengers and workers spoke of their own safety being dependent on the behaviour of others.

*“The biggest bane in our lives is we can’t enforce, and not to be confrontational. So a driver could turn round and say to someone, have you got a face covering/a mask? And if they say no, that’s it, there’s nothing they can do.” (W6, Phase 1)*

*“I think the bus company management need to give their staff more back up, and make it clear that there will be enforcement. I think the train guards need to, again, the few people who aren’t wearing masks, make it clear that they will be turfed off at the next station if they don’t comply.” (P8, current passenger, Phase 1)*

This issue of enforcement powers was still a consideration for respondents in Phase 2. During Phase 2, the mandate for wearing face coverings on public transport was removed, and this presented a different difficulty for some companies. Organisational leaders explained that without the government mandate for face coverings, it was not possible for the organisation to ‘require’ face coverings for any employees or passengers, without legislative backing. This was reported to cause challenges for organisational leaders where there were workers or passengers who wanted the mandate for face coverings to continue.

By Phase 2, in addition to compliance, issues of behaviour and discipline fatigue were starting to be discussed more. People were viewed as tiring of control measures, and it was also suggested that people had become habituated to the perception of risk.

*“Behavioural barriers are part of the compliance issue. [ … ] People have become quite tolerant of some of these risks and actually it’s quite difficult in some scenarios to keep people apart, and that’s difficult and frustrating for them, for their supervisors.” (EX1, Phase 2)*

*Capacity and crowding measures*

Advice to the public about making journeys changed throughout the pandemic; therefore, it was consistently difficult for operators to predict what demand would be on particular services, and thus to plan capacity. Additionally, crowding was also affected by other unpredictable factors such as lock downs, weather, changes to peak travel patterns, and service cancellations. This presented challenges for organisational leaders in terms of planning staffing and capacity.

Capacity restrictions also limited revenue for companies, in addition to costs of changes to operations to implement COVID-19 mitigations.

*Staffing; test and isolate*

Many of the organisational leaders and experts suggested that work absence became more challenging during the Omicron wave (Dec 2021). They had previously struggled with the ‘pingdemic’ in Autumn 2021 (forced isolation due to close contact with someone who had tested positive), but more workers were ill during the Omicron wave. While some of the larger organisations had set up and / or considered formal staff testing (Phase 1), most had encouraged regular testing (lateral flow) but not made arrangements compulsory. There were a few exceptions for critical roles such as control room staff or those engaged in training drivers where testing was compulsory due to essential staff and close proximity. Free testing was available to the public during both Phases of the research, but was withdrawn from April 2022.

*Lack of transmission data on public transport*

Some experts highlighted the lack of data around transmission and transmission routes, and wanted more evidence to be collected and made available.

*“I guess the professional answer is that we don’t have good data to indicate either way, but there are some of the same risk factors in place for transport which we’d expect to be in place for a high risk environment, which would be a number of people in close proximity potentially without the ability to physically distance in an enclosed space for an extended period of time.” (EX3, Phase 1)*

##### Facilitators

*Industry cooperation*

During Phase 1, the main facilitators related to cooperation, communication and clear messaging. Where service providers and companies worked with others in the industry and actively supported and listened to workers, this cooperation was found to aid the development, communication and implementation of mitigation measures.

Additional facilitators included important innovations such as the development of COVID-19 tests and vaccines, as well as technological innovations to working practices such as ticket purchasing, assessing capacity, and ordering apps.

*Low passenger numbers*

It was widely recognised that low passenger numbers were helpful in reducing the risk of transmission on public transport, but also that it was difficult to know how the situation would change with increased numbers. Many respondents spoke about the increased importance of mitigations when this happened.

*“It's going to be an enormous challenge, because I mean at the moment it's easy because there's nobody travelling on the network, so it's very easy to keep it clean, for people to socially distance themselves and so on.” (OL1, Phase 1)*

*Technology*

Companies used technology in a range of ways to address challenges. Some developed apps for passenger purposes such as cashless travel (or modified collection mechanisms), at-seat ordering, managing seat reservations, or checking busyness of services. Technology was used increasingly with staff for communication and training, maintaining engagement with managers, and create online spaces where colleagues could meet and socialise.

*COVID tests and vaccines*

The development of COVID-19 tests and vaccines and the availability of home lateral flow tests were reported to be essential tools for managing viral transmission. While isolation periods were facilitated for workers, reductions in the duration of isolation time for positive COVID cases and contacts were reported to have made a significant difference to the maintenance of operations. The reduced duration of isolation meant staff could return to work sooner, and where contacts could take daily lateral flow tests instead of isolating meant that they did not need to leave the workplace at all. Therefore, the availability of (free) lateral flow tests and related isolation periods were viewed as important tools for continued service.

*“The one thing that’s really helped is the changes in the self-isolation guidance, and the widespread availability of lateral flow testing. I mean, in many ways, lateral flow testing is what’s kept us at work, ‘cause we’ve been having people do two lateral flow tests a week.” (OL4, Phase 2)*

### NOW WHAT could be improved and used to respond better in future?

The respondents provided a number of helpful reflections on ways in which the changes to manage viral transmission on public transport were implemented.

#### Things that went well

##### Enhanced sector cooperation

Cooperation and communication within the sector has been important, particularly in the early stages of the pandemic (Phase 1). However, it has remained a useful facility for sharing best practice in the later stages (Phase 2). It appears that this was most useful when changes were being brought in quickly and consistent information was difficult to source. Maintaining effective co-operation / relationships will be essential to any future timely cross sector response to future emergencies.

*“Because it was done at such a senior level, they could have pretty honest conversations with each other. But because it was also private, it meant that they weren’t having to defend their position to their members or the government, or whoever. It was a good space we created and I think we created a very useful working environment there.” (EX18, Phase 2)*

##### Development of a ‘guidebook’ of lessons that can be used for future waves of COVID-19 or other public health emergencies

By Phase 2, it was clear that much had been learnt about the management of SARS-CoV-2 within the public transport sector. Experts and organisational leaders had built up knowledge and experience and spoke about having a ‘toolbox’ of strategies that they could use when necessary, and that this saved time in implementation. There was also a sense that these skills and tools should be well documented now in order to solidify the learning and make it available if it is needed in the future. For such a resource to be most effective in the future, the industry needs to be aware of its contents and applicability, as well as how it can be accessed.

*“The view is now that really we want a playbook of just things we can pull out and we want to know what the deal is without having to go through a huge faff and a huge bit of confusion and last minute policymaking. That's what people want now.” (EX3, Phase 2)*

*“I think at various points we've made some mistakes, some slightly naïve decisions and some quite big lessons we learnt off the back of it. So that's helped as we’ve gone into this Omicron wave, but I think it will probably stand us in better stead as we go into anything that might come up after this as well.” (OL18, Phase 2)*

#### Issues that arose and need improving for any future emergency

##### Communication

Communications with government, and direct communication from government to passengers, caused some difficulties within the sector. These issues were raised by many at Phase 1; by Phase 2 communications from government were less frequent but the continued impact of some of the early messaging was still being felt.

One of the main points of reflection for respondents was on the communication from the government, through two channels: first, direct government communication with the public regarding public transport; second, communication from government to the public transport industry. Consistent messaging was the most commonly suggested improvement, to reduce confusion and improve compliance.

*“One of the first things that a new manager learns, management 101, communication. Have a consistent communication, communicate your message clearly and simply and be consistent, don’t chop and change your position. My goodness we have chopped and changed our position in terms of COVID up and down these islands.” (OL12, Phase 2)*

Furthermore, experts and organisational leaders reflected on the reduced passenger numbers and suggested that a concerted effort would be required in order to increase numbers, and that specific and direct communication with the public would be required to achieve this aim.

##### Leadership

The role of leadership in the oversight and daily management of public transport through the pandemic was discussed as being very important by respondents. This factor relates strongly to the need for clear and consistent communication during an emergency, but respondents also suggested good behavioural examples set by senior members of staff impact on the behaviours of others.

*“But, when, perhaps a more senior person was one of the ones that was temporarily lowering their mask, it’s not for me to question to the person responsible for the train, i.e. the train manager, would you mind pulling your mask up? It’s difficult.” (W5, Phase 1)*

##### Behavioural compliance

Adherence with mitigations by workers and passengers was of central importance. At Phase 1, compliance was a significant issue in the perception of transmission risk within the public transport sector. Examples of non-adherence were reported by all respondents, and negatively affected perceptions of risk and caused interpersonal tensions. By Phase 2, tensions had eased, but compliance still affected perceptions of risk. There was also a growing appreciation of the complexity of these behaviours across different contexts.

Clear communication about accountability regarding adherence was also lacking**.** Workers and passengers held differing beliefs about the ability to ‘enforce’ rules. This was very clear at Phase 1, but at Phase 2, passengers were still asking for workers to be given more ‘authority’ to refuse entry to those not adhering to the face covering mandate. In future, making clear where the responsibility for ‘enforcement’ lies, and the legislative powers associated with this responsibility, will reduce the divergence in understanding between groups.

*“When [the government] asked us actually about exemptions, we said, you know, better not to make people exempt, or make people have to wear something or show something quite visible, and they decided not to go down that route. But then they still expected us to enforce face coverings which was really very difficult to do. So yeah, there was definitely a lack of consultation and a lack of understanding of how to actually implement some of what they were instructing.” (EX2, Phase 1)*

##### Decision making power

Some organisations reported maintaining mitigations including advice to wear face coverings (for both passengers and staff) when the government mandate was removed. However, they also reported in Phase 2 that they lacked authority to maintain/enforce mitigations after removal of government regulations.

Other organisations felt they could not maintain mitigations and that that their hands were tied.

*“There’s been quite a number of colleagues who are very vociferous about the removal of the requirements for face coverings in the summer [2021], and felt we should continue to mandate them, and that we were putting them at risk, because the passengers weren’t wearing face coverings. [ … However] it really wasn’t enforceable, once there was no legal requirement, there was nothing we could do, which some of our colleagues found very difficult, but others really weren’t too bothered.” (OL4, Phase 2)*

Decision making within organisations was also difficult when employee opinion was divided on issues of mitigation. Joint decision making with employees caused some issues because some people felt safe while others did not, and it was hard to balance the needs of both groups under changing circumstances. Careful consideration of how organisations should go about this in the future is required.

## Discussion/recommendations

**Fig. 2:**
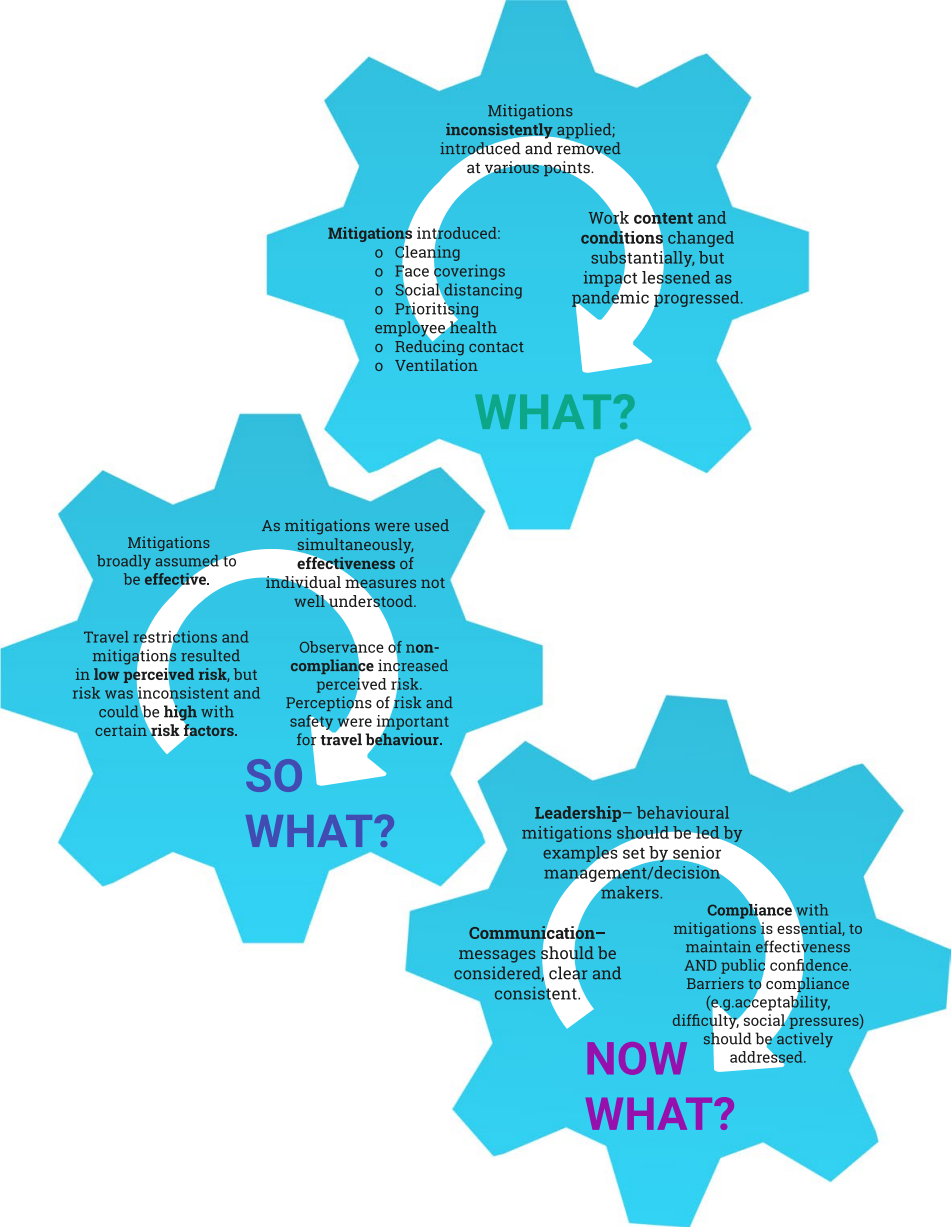
Reflections on the COVID-19 pandemic within the public transport sector

Taking a reflective approach on a variety of stakeholder voices, this study has characterised what happened through the course of the pandemic, what the implications of these actions were, and how pandemic management on public transport could be improved [19, 20]. This approach was designed to lead to suggestions for the management of future public health emergencies in the public transport sector. The importance of learning for the future was stressed by many of the respondents (experts and organisational leaders). The longitudinal approach allowed us to capture changes in policy and mitigation measures as well as changing risk perceptions within the fast moving nature of the pandemic, and to observe how this developed over time. Figure 2 provides a summary of our reflective process and findings.

There has been limited research directly related to management of SARS-CoV-2 transmission risk on public transport. Early work looking at the risk of transmission in this setting was reviewed by Gartland et al. [27], and highlighted a number of gaps in knowledge at the time including: relative importance of transmission routes, effectiveness of controls across modes of transport, what factors affect compliance with behavioural mitigations, and transmission risk on public transport compared to other activities.

In an early study of passenger opinion, travellers’ views of risk and mitigation was carried out through a rail route choice task by Shelat et al. [28]; this cross-sectional study was conducted in December 2020, and showed that vehicle crowding and infection rates were the primary factors informing risk perception, and face covering mandates were perceived as the most important mitigation measure. Train travellers also valued increased sanitisation on journeys. These findings are consistent with our findings, particularly from Phase 1, and showed a particular appetite for visible mitigations among passengers at this time. A separate study from Brazil (July 2020) also confirmed the findings from passengers in the present study that crowding was viewed as a significant risk factor by this group [29].

Looking more widely at research conducted outside the context of public transport, many aspects of the approach to pandemic management in public transport have been researched. While the effectiveness of some individual mitigations may be debated [30, 31], it is important to recognise the value of having multi-layered mitigations in place [32–35]. Furthermore, when considering the effectiveness of particular interventions, it is important to consider any unique features of the context. For example, evidence suggests that face covering compliance was higher on public transport than in workplaces and leisure settings [36]. Higher levels of compliance will increase the effectiveness of face coverings to reduce transmission. This may be explained by context-dependent acceptability; evidence of travel choices from Switzerland indicated that the introduction of a ‘mask mandate’ did not affect ridership levels [37], suggesting that such mandates were acceptable to passengers. Finally, it is important to consider the social role of certain mitigations, which may work separately to the actual impact on viral transmission, but can influence passengers perceptions of safety [38].

There is strong evidence accumulating for the benefits of ventilation on public transport [39, 40]. However, some other mitigations such as plastic screens and face visors, and cleaning practices, including hand sanitising and surface treatments, may not have a strong impact on SARS-CoV-2 transmission [30, 41]. Haug et al. [41] question the effectiveness of social distancing on public transport, although the authors acknowledge that this may be due to an increased likelihood that people wear face coverings in this context. Others found that many of the non-pharmaceutical interventions implemented in a wide range of countries may not have reduced Covid-19 fatalities [31].

Balancing the findings of the reflective process, six recommendations were drawn:

1. **Maintain cross-industry cooperative working practices**.

The sector found some of the decision-making and quick implementation of changes challenging. The collaborative links and fora established within the public transport sector during the pandemic now present a point of contact for joint decision making both with and across the industry. The trust built through these fora could facilitate consultation with the industry as a whole, as well as promote collaborative effort to make decisions and implement change. The maintenance of these links will require a proactive approach from all stakeholders.

2. **Access to good quality knowledge, information and data through industry or governmental networks is necessary.**

Evidence-based practice is a fundamental principle in health and medicine, and where possible, public health decisions should be aligned to valid risk data and research evidence. During a pandemic, there are challenges to the availability of this novel information, but it is essential that decisions are based on the best data and research evidence available, and that decisions are reviewed in concert with new evidence. Some information was reported to be difficult to source at the start of the pandemic (such as technical details of mitigations and routes of transmission), and some information (such as data recording transmission of COVID-19 on public transport) has never been available. Such information would aid understanding of the risks associated with different settings and allow mitigations to be implemented proportionately. The availability of detailed and accurate information will also aid organisations in conducting accurate risk assessments, which are essential, especially when organisations are required to manage their own risks (i.e. when regulations are not given by government). Consideration for collecting relevant data, it’s monitoring and usage in future will be important, especially where threats to public health are long lasting.

3. **Better understanding of mitigation effectiveness in the context of public transport.**

There is a particular gap in knowledge around the combined effectiveness of the mitigations implemented to reduce transmission on public transport. Evidence suggests that the following mitigations are effective in reducing the risk of SARS-CoV-2 transmission: ventilation, reducing crowding, wearing face coverings, and vaccination [30]. However, there is still limited understanding of the combined effects, though early evidence from a public health perspective suggested that it was indeed the power of combined mitigations that slowed the spread of the virus [41]. Research to quantify this would aid future decision making, but may need to draw on transmission data from other settings given the limited evidence available pertaining directly to public transport. However, the context needs to be carefully considered; for example, evidence suggests that compliance with face covering rules was higher on public transport compared to workplaces and leisure settings [36]. In addressing issues of viral variants, it would be helpful to understand the viral parameters within which different mitigations or combinations of mitigations are effective, such as level of transmissibility or primary route of transmission. For public transport, social distancing has a fundamental impact on operations and revenues. Given these challenges, it is essential to understand what the removal of this mitigation means for other mitigations such as face coverings, hand hygiene and ventilation (e.g. how well do these work if social distancing is not in place? Are they *more* important without social distancing?).

4. **Improved messaging and communication**.

Collaborative development of clear and timely messages and communications should be considered. This would focus on the avoidance of mixed messaging. Where possible, consistency in messages and rules between devolved countries and across different modes of transport would help with clarity as well as compliance and enforcement for both workers and passengers.

Issues with messaging and communication were not constant across the pandemic. At Phase 1, issues arose mainly from ambiguities in the communication of guidance from government, which was seen as a result of the fast moving pace of change at this stage. However, by Phase 2, issues of understanding were more prominent, which was related to the messaging changing frequently and mitigations being implemented, removed and re-implemented. Guidance from behavioural scientists should be followed to promote understanding and compliance with public health messages [42], and also to better understand the social psychology of visible behavioural mitigations such as wearing face coverings [38].

5. **Reliance on behavioural mitigations is complex.**

Compliance with behavioural mitigations was one of the key issues raised during both phases of data collection, with a range of different barriers to behaviour identified. Given the centrality of behavioural mitigations to the approach to the COVID-19 pandemic, lessons have been learnt about the challenges and limitations of changing people’s behaviour. If behavioural mitigations were required again, It is essential to understand the motivations for complying with behavioural mitigations under a range of circumstances for behaviour change to be successful. It is also important to appreciate that behavioural compliance is not static and it is influenced by time-varying factors such as risk habituation and behavioural fatigue. Research into the drivers of these behaviours (in a variety of contexts) could help identify interventions to enhance future compliance with mitigations.

6. **Clear lines of accountability are important.**

The lines of accountability for dealing with issues of non-compliance with guidance for both passengers and workers could be clarified. The passengers in the present study differed from all other stakeholders in their understanding of this. Passengers suggested that frontline public transport workers should have the responsibility of maintaining adherence to COVID-19 guidance through encouragement, as well as with enforcement powers for relevant COVID-19 regulations. This difference in understanding caused frustration for both passengers and workers. Careful consideration of the how mandates are implemented, and communicated to passengers and workers, could include: explicit definition of where responsibility for compliance lies, clarification of any lines of accountability, and transparency in enforcement roles (or lack thereof) across different mitigations.

In addition to the recommendations specific for the public transport sector outlined here, several cross-sector recommendations can also be made on the basis of the current findings.

First, interpersonal workplace dynamics should be considered when implementing behavioural mitigation measures as the nature of relationships between colleagues and with the public will affect adherence. General government guidance advising the avoidance of contact with people outside your immediate household was successfully implemented in many contexts in the public transport sector; in particular, this was an observed strategy with strangers and/or passengers. However, the guidance was less frequently followed with colleagues. The level of familiarity and trust between some co-workers made the implementation of social distancing, cleaning and face coverings challenging in a way that was not mirrored in their contact with the general public. To address this, additional information, guidance and messaging focussing on colleague interactions, such as during breaks, work at depots / stations and travel to work, could improve compliance with behavioural mitigations between colleagues. Behavioural and occupational scientists have the expertise to research and understand these context-dependent behaviours and should be consulted to help word and deliver messaging to raise compliance in all circumstances [43].

Second, the importance of leaders in creating safe workplace COVID-19 cultures should not be underestimated. Managers at all levels, and especially immediate line managers need to be aware of the impact of their behaviours on likely compliance in their teams.

Third, support for organisational decision-making should be considered. The removal of regulations meant that it was not feasible to keep effective mitigations in place that would have made workers feel more comfortable regarding transmission risks. In order to improve decision-making power within organisations in the future, organisations require clear communication about decisions regarding public health regulations relevant to public transport, how such decisions are reached, and who is accountable for the decisions. Where organisations are required to make their own decisions about mitigation, detailed, clear and accurate information about risks is necessary in order for risk assessments to aid decision making across contexts.

At the time of writing, society has moved on again; and is now in the phase of ‘Living with COVID-19’ [44], in which there are no public mitigations in place on public transport in the UK. However, future waves of COVID-19 or an alternative novel respiratory virus are possible, and could have the potential to lead to the re-introduction of mitigation measures. It is also important to consider the wider context, and the developments since this data was collected. Levels of ridership on National Rail and buses have recovered somewhat, but are still notably short of pre-pandemic levels; in March 2023 this stood around 93% for National Rail and around 90% for buses, with ridership lower on London buses than for the rest of the country [45].

In conclusion, the wealth of experience gained through the COVID-19 pandemic in the public transport sector is extremely valuable. This paper has captured useful lessons learned in the public transport sector using reflective practice to explore what happened over the course of the pandemic, what it meant, and how the pandemic response could be improved. The recommendations made on the basis of this process will help to improve public health strategy within the public transport sector.

## Data Availability

The datasets generated and analysed during the current study are not publicly available in order to protect participant confidentiality, and consent was not sought from the participants to make this qualitative data publicly available but data are available from the corresponding author on reasonable request.

## Abbreviations

CPT: Confederation for passenger transport
DfT: Department for transport
EX: Experts
HSE: Health and safety executive
LAs: Local authorities
OL: Organisational leaders
ONS: Office for national statistics
ORR: Office of rail and road
P: Passengers
PCR: Polymerase chain reaction
PHV: Private hire vehicle
PPE: Personal protective equipment
RDG: Rail delivery group
REACT: Real-time assessment of community transmission
RSSB: Rail safety and standards board
UKHSA: United Kingdom health security agency (formerly Public Health England, PHE)
W: Workers

## Footnotes

1 In this publication, there is a focus on lessons learned that are specific to public transport. However, wider lessons were also drawn out in the original reports of the research [8, 9].
